# Supplementary material for: Blind source separation of event-related potentials using a recurrent neural network
Source: bioRxiv. 2024 Apr 28:2024.04.23.590794. Preprint. [Version 1] doi: 10.1101/2024.04.23.590794 (PMC11071372; doi:10.1101/2024.04.23.590794)
Supplement: Supplement 1 [file NIHPP2024.04.23.590794v1-supplement-1.pdf]

bioRxiv preprint doi: https://doi.org/10.1101/2024.04.23.590794; this version posted April 28, 2024. The copyright holder for this preprint (which was not certified by peer review) is the author/funder, who has granted bioRxiv a license to display the preprint in perpetuity. It is made available under aCC-BY-NC-ND 4.0 International license.

Supplementary Information

Table S1 Analysis of RNN and ICA sources

Source	*MMN (r)*	*N170 (r)*	*N400 (r)*	*P3 (r)*	*MMN (MSE)*	*N170 (MSE)*	*N400 (MSE)*	*P3 (MSE)*	*SE*	*Most similar*
RNN 01	0	0.101	0	0.868	0.078	0.493	0.212	0.197	0.334	ICA01
RNN 02	0.179	0.746	0	0.166	0.076	0.24	0.212	0.75	0.843	ICA04
RNN 03	0	0.368	0.042	0.509	0.078	0.464	0.211	0.588	0.834	ICA02
RNN 04	0.269	0.136	0.889	0	0.074	0.489	0.046	0.768	0.822	ICA02
RNN 05	0	0.554	0	0	0.078	0.363	0.212	0.768	0	ICA04
RNN 06	0.87	0.24	0.301	0.112	0.02	0.469	0.195	0.76	1.124	ICA04
RNN 07	0	0.464	0	0	0.078	0.412	0.212	0.768	0	ICA05
RNN 08	0	0	0	0.205	0.078	0.496	0.212	0.741	0	ICA17
RNN 09	0	0	0	0.2	0.078	0.496	0.212	0.74	0	ICA15
RNN 10	0	0.206	0	0	0.078	0.48	0.212	0.768	0	ICA06
RNN 11	0	0.199	0	0	0.078	0.478	0.212	0.768	0	ICA19
ICA 01	0.36	0.286	0.488	0.794	0.072	0.464	0.171	0.444	1.31	RNN01
ICA 02	0.606	0.468	0.8	0.6	0.066	0.464	0.152	0.642	1.368	RNN04
ICA 03	0.063	0.4	0.104	0.647	0.078	0.471	0.21	0.686	1.064	ICA01
ICA 04	0.816	0.813	0.429	-0.027	0.048	0.315	0.184	0.786	1.116	RNN02
ICA 05	0.468	0.603	0.473	0.268	0.072	0.457	0.201	0.752	1.349	ICA04
ICA 06	0.491	0.439	0.326	0.242	0.073	0.439	0.203	0.737	1.352	ICA10
ICA 07	0.486	0.508	0.186	0.292	0.071	0.458	0.206	0.716	1.315	ICA08
ICA 09	0.154	0.229	0.61	0.184	0.077	0.485	0.185	0.75	1.215	ICA02
ICA 10	0.141	0.068	0.445	0.381	0.077	0.494	0.19	0.74	1.181	ICA02
ICA 11	0.205	0.156	0.054	0.373	0.076	0.491	0.211	0.741	1.21	ICA06
ICA 12	0.367	0.441	0.145	0.088	0.076	0.482	0.209	0.765	1.215	ICA04
ICA 13	0.007	0.104	0.261	0.346	0.078	0.494	0.208	0.76	1.047	ICA01
ICA 14	0.179	0.344	0.213	0.029	0.077	0.465	0.207	0.767	1.18	ICA04
ICA 15	0.096	0.238	-0.068	0.304	0.078	0.484	0.213	0.753	1.226	ICA03
ICA 16	0.325	0.186	0.141	0.213	0.075	0.487	0.208	0.745	1.339	RNN09
ICA 17	0.342	0.265	0.07	0.16	0.075	0.485	0.211	0.758	1.253	ICA06
ICA 18	0.022	0.009	0.187	0.262	0.078	0.496	0.21	0.752	0.911	RNN08
ICA 19	0.386	0.221	0.21	0.104	0.076	0.492	0.21	0.765	1.29	ICA07
ICA 20	0.051	0.047	0.298	0.33	0.078	0.495	0.205	0.758	1.089	ICA09
ICA 21	0.023	0.118	0.074	0.163	0.078	0.491	0.211	0.761	1.215	ICA01
ICA 22	0.183	0.198	-0.005	0.101	0.077	0.492	0.212	0.764	1.107	ICA19
ICA 23	0.09	0.199	0.052	0.034	0.078	0.488	0.211	0.767	1.17	ICA14
ICA 24	0.061	0.11	0.13	0.086	0.078	0.494	0.21	0.765	1.349	ICA14
ICA 25	0.191	0.13	0.105	0.067	0.077	0.491	0.21	0.764	1.319	ICA02
ICA 26	-0.009	0.23	0.153	-0.002	0.078	0.491	0.21	0.769	0.802	ICA06
ICA 27	0.074	0.055	0.072	0.05	0.078	0.495	0.211	0.766	1.371	ICA14

Sources from each method are ranked from highest to lowest by overall correlation of their projections across four ERP difference waveforms

Correlation (r) and MSE were calculated from projections of source signals onto scalp sensors, comparing against ground truth grand-average ERP difference waveforms

Shannon entropy (SE) of each source was calculated from its correlations with the four ERP difference waveforms

Most similar source was that with the maximum similarity score (SS) calculated using equation (7)

bioRxiv preprint doi: https://doi.org/10.1101/2024.04.23.590794; this version posted April 28, 2024. The copyright holder for this preprint (which was not certified by peer review) is the author/funder, who has granted bioRxiv a license to display the preprint in perpetuity. It is made available under aCC-BY-NC-ND 4.0 International license.

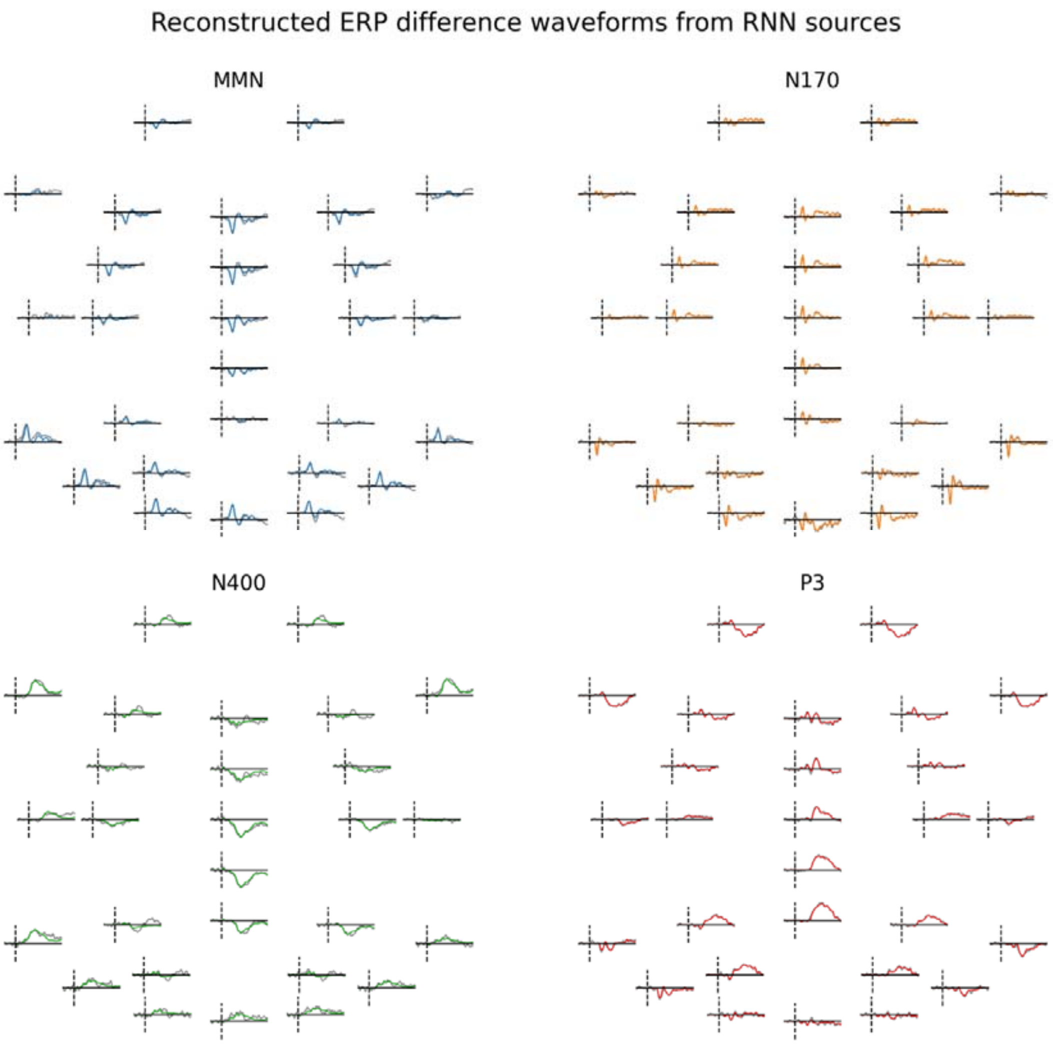

Figure S1 Reconstructed difference waveforms from RNN sources plotted at 28 EEG channels. Reconstructed waveforms are plotted in colour and ground-truth waveforms are plotted in black, although two traces on most plots are difficult to distinguish because they are overlapping. All of the electrodes in each quadrant are plotted with the same y-axis scale.

bioRxiv preprint doi: https://doi.org/10.1101/2024.04.23.590794; this version posted April 28, 2024. The copyright holder for this preprint (which was not certified by peer review) is the author/funder, who has granted bioRxiv a license to display the preprint in perpetuity. It is made available under aCC-BY-NC-ND 4.0 International license.

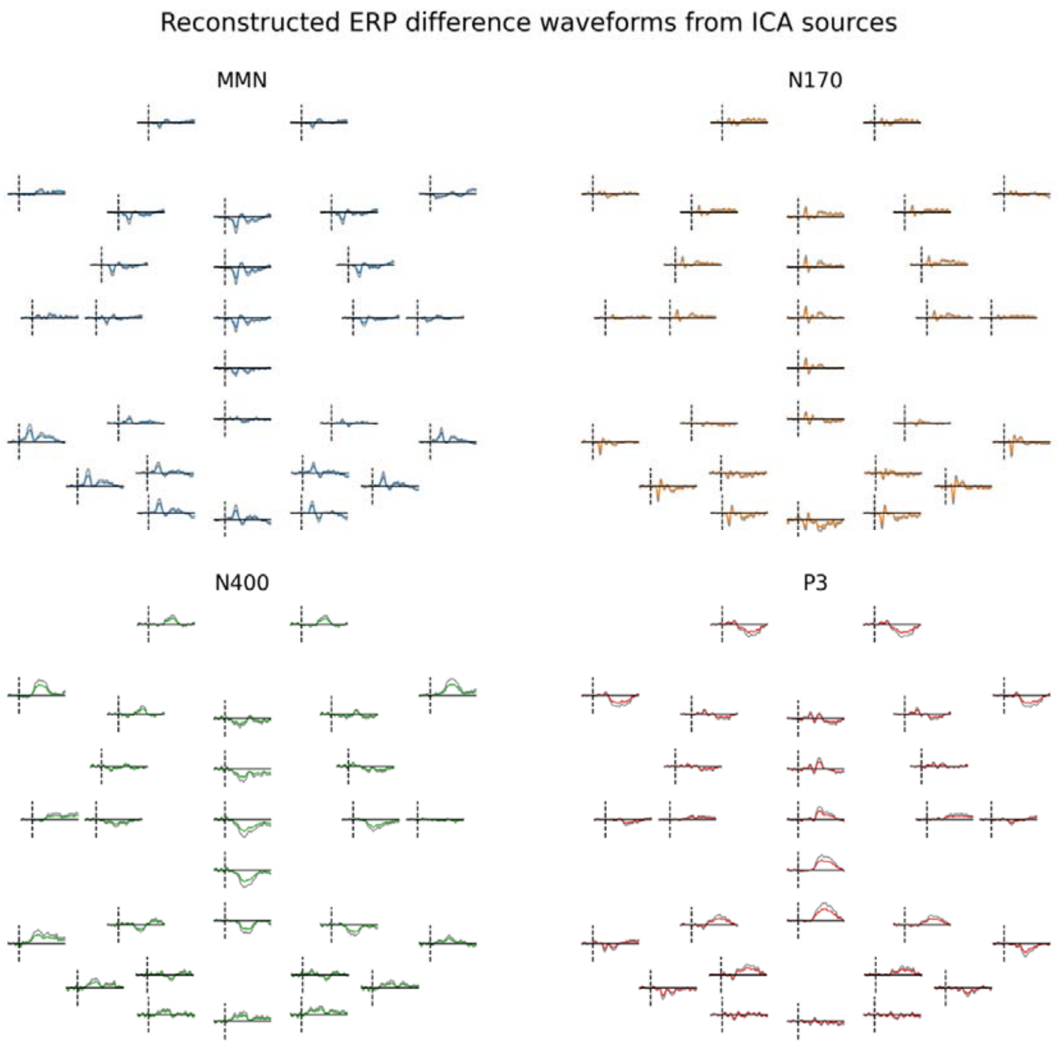

Figure S2 Reconstructed difference waveforms from ICA sources plotted at 28 EEG channels. Reconstructed waveforms are plotted in colour and ground-truth waveforms are plotted in black. Y-axis scales are the same for each electrode in the montage, but different for different ERP difference waveforms (MMN, N170, N400, and P3). These reconstructed waveforms have higher MSE compared with those from RNN sources, but otherwise are comparably well-correlated with ground-truth waveforms.

bioRxiv preprint doi: https://doi.org/10.1101/2024.04.23.590794; this version posted April 28, 2024. The copyright holder for this preprint (which was not certified by peer review) is the author/funder, who has granted bioRxiv a license to display the preprint in perpetuity. It is made available under aCC-BY-NC-ND 4.0 International license.

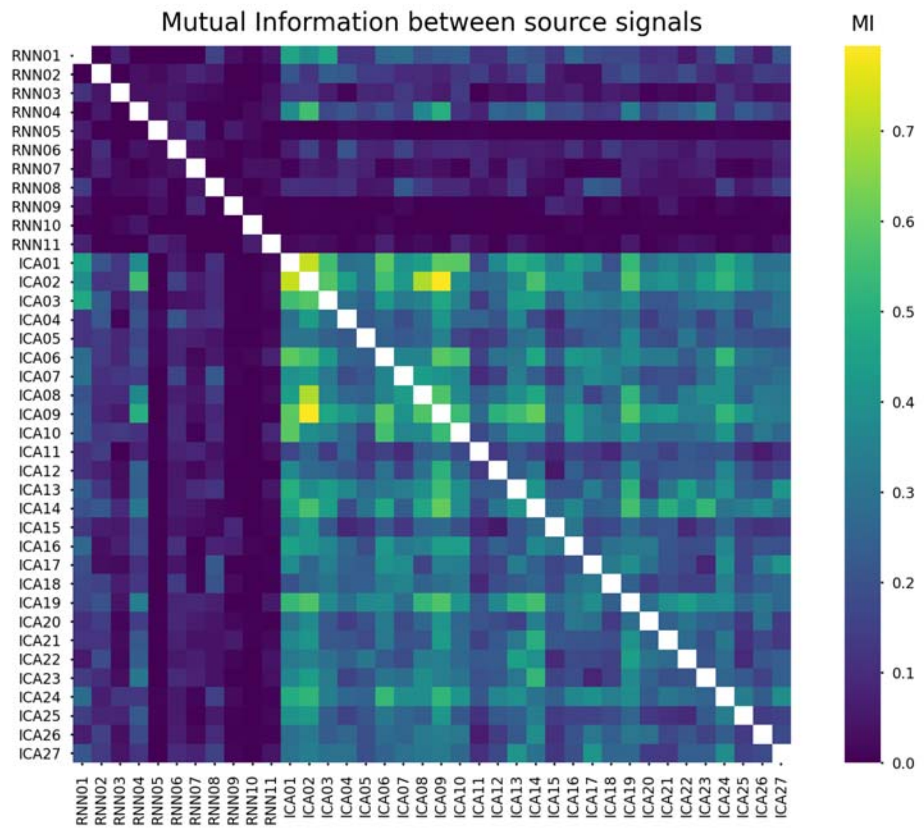

Figure S3 Pairwise mutual information between RNN and ICA sources. MI is generally higher between ICA sources than it is for RNN sources. There also tends to be higher MI between RNN and ICA sources than within RNN sources. These dependencies are reflected by signal waveforms for these sources.

bioRxiv preprint doi: https://doi.org/10.1101/2024.04.23.590794; this version posted April 28, 2024. The copyright holder for this preprint (which was not certified by peer review) is the author/funder, who has granted bioRxiv a license to display the preprint in perpetuity. It is made available under aCC-BY-NC-ND 4.0 International license.

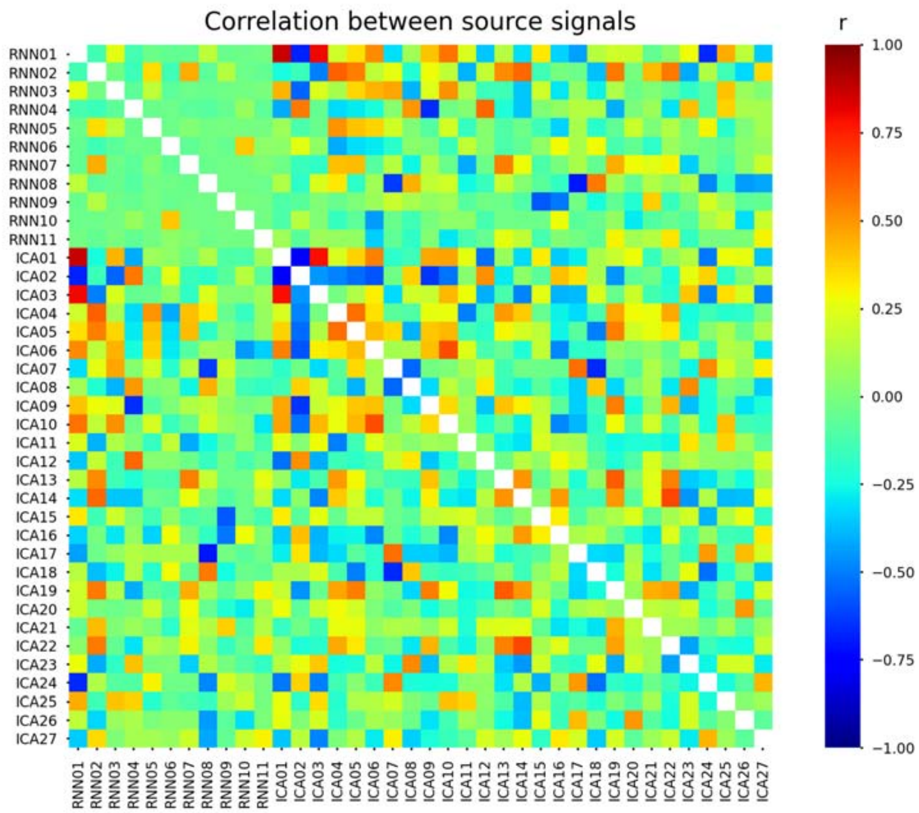

Figure S4 Pairwise correlations between signals for each RNN and ICA source. A similar pattern seen from MI analysis is observed for correlation between source signals. However, signals can be anticorrelated because ICA allows positive and negative source amplitudes, which may reflect inversions of RNN sources. Correlations within ICA sources are higher than those within RNN sources, and RNN sources tend to have greater correlations with ICA sources than with RNN sources.

bioRxiv preprint doi: https://doi.org/10.1101/2024.04.23.590794; this version posted April 28, 2024. The copyright holder for this preprint (which was not certified by peer review) is the author/funder, who has granted bioRxiv a license to display the preprint in perpetuity. It is made available under aCC-BY-NC-ND 4.0 International license.

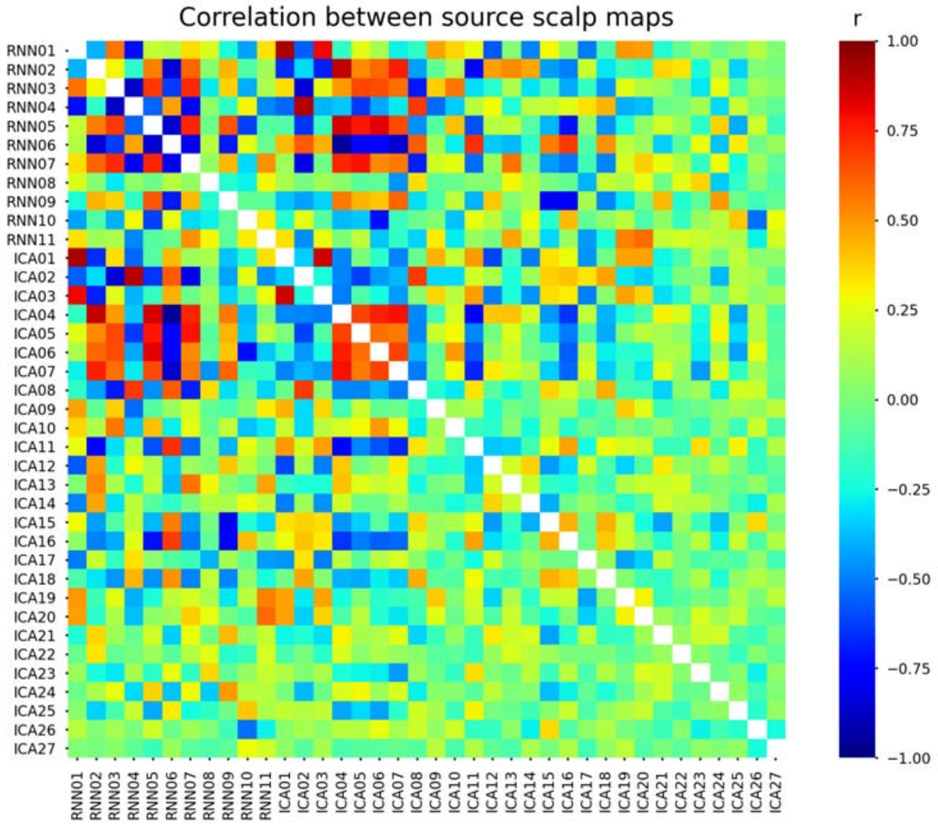

Figure S5 Pairwise correlations between scalp maps for each RNN and ICA source. There are high correlations within and between RNN and ICA sources, reflecting similarity of scalp distributions. For RNN sources, negative correlations indicate opposite polarity contributions to scalp potentials. However, for ICA sources this relationship is ambiguous because ICA source signals can have biphasic polarity.

bioRxiv preprint doi: https://doi.org/10.1101/2024.04.23.590794; this version posted April 28, 2024. The copyright holder for this preprint (which was not certified by peer review) is the author/funder, who has granted bioRxiv a license to display the preprint in perpetuity. It is made available under aCC-BY-NC-ND 4.0 International license.

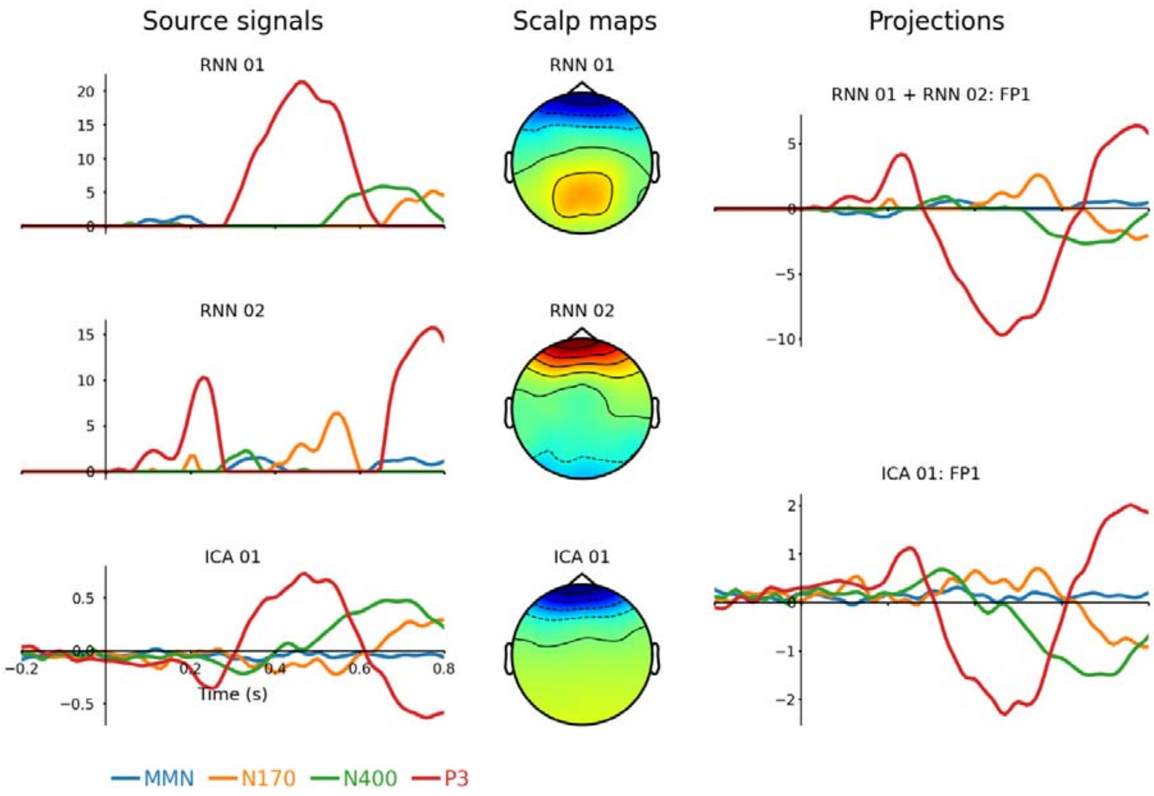

Figure S6 Example of an eye-blink source represented by RNN and ICA methods. This analysis was performed on data before correcting eye-blink artifacts. Source ICA 01 captures both phases of activity caused by changes in electric dipole orientation as the eye articulates in its socket while blinking. RNN 01 captures negative portions and RNN 02 captures positive portions of the blink artifact; positive and negative polarities given in terms of scalp projections at electrode FP1. The summed projections from RNN 01 and RNN 02 are highly correlated with the projection from ICA 01. Source waveforms and projections are plotted on the same time range. Differences in the latency of this systematic eye-blink artifact for N170, N400 and P3 may reflect differences in stimulus duration and interstimulus-interval (ISI) in each of these paradigms (i.e., N170: 0.3 s duration, 1.1-1.3 s ISI; N400: 0.2 s duration, 0.9-1.1 s ISI before target words; P3: 0.2 s duration, 1.2-1.4 s ISI).

bioRxiv preprint doi: https://doi.org/10.1101/2024.04.23.590794; this version posted April 28, 2024. The copyright holder for this preprint (which was not certified by peer review) is the author/funder, who has granted bioRxiv a license to display the preprint in perpetuity. It is made available under aCC-BY-NC-ND 4.0 International license.

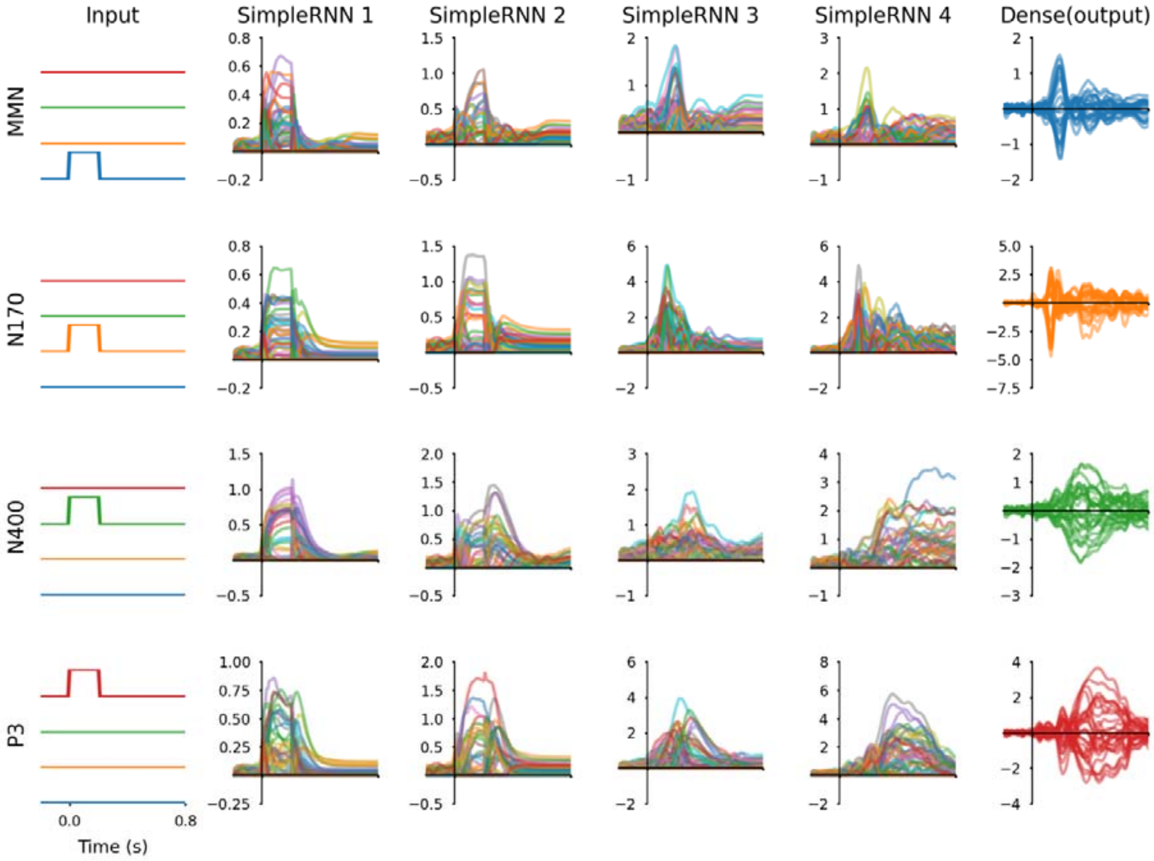

Figure S7 Inputs, hidden unit activations, and output signals from the RNN after training phase 1 (without L1 regularization applied to *SimpleRNN* 4 layer). All 64 hidden units from SimpleRNN 4 are involved in producing outputs that match ERP difference waveforms; this makes it possible to use the RNN method to separate more sources than the number of EEG channels. All waveforms are plotted from −0.2 s to 0.8 s about stimuli onsets that occurred at 0.0 s. Hidden unit activation waveforms have arbitrary units and output waveforms have microvolt units.

bioRxiv preprint doi: https://doi.org/10.1101/2024.04.23.590794; this version posted April 28, 2024. The copyright holder for this preprint (which was not certified by peer review) is the author/funder, who has granted bioRxiv a license to display the preprint in perpetuity. It is made available under aCC-BY-NC-ND 4.0 International license.

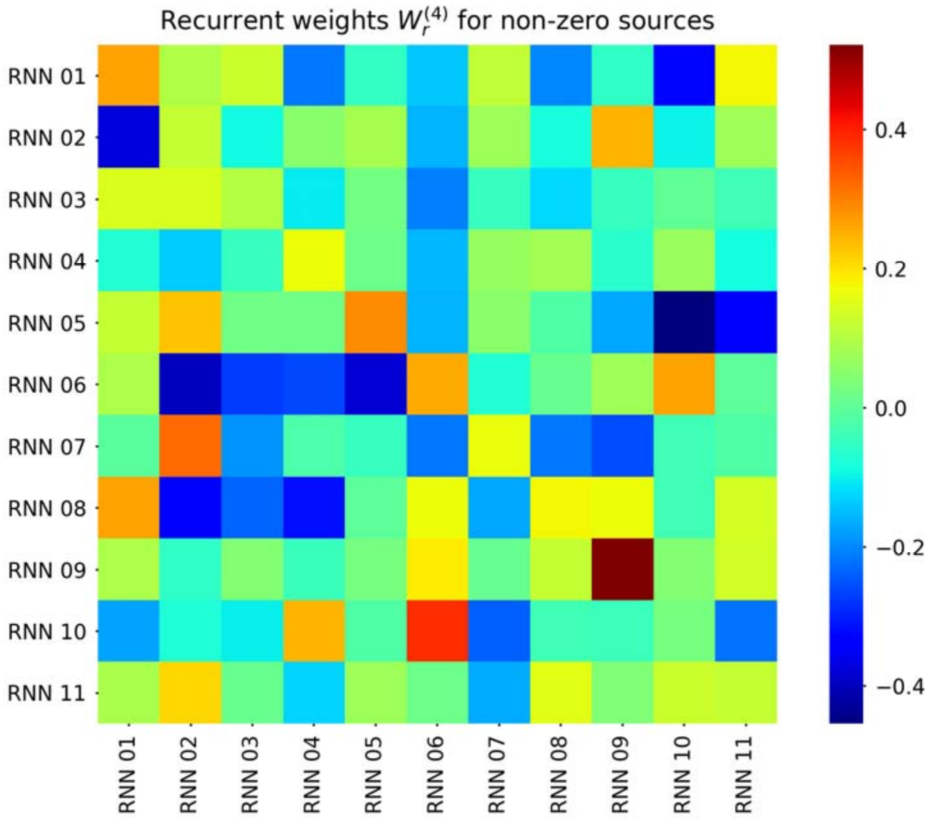

Figure S8 Recurrent weights from the *SimpleRNN 4* layer of the RNN. These recurrent weights determine the influence of *affector* source at time in each row with *affected* source at time in each column; hence the direction of influence is from row to column, as presented in this figure. Negative values indicate the tendency for an affector source to diminish or suppress activity of the affected source, while positive values indicate a tendency for an effector source to enhance or induce activity of the affected source. These recurrent weights thus reflect functional connectivity among sources. It is important to remember that inputs from the preceding hidden layer (*SimpleRNN* 3) also influence the activity of these sources.
